# Determination of different social groups’ level of knowledge about malaria in a multicultural Amazonian cross-border context

**DOI:** 10.1186/s12889-023-16507-9

**Published:** 2023-08-19

**Authors:** Mélanie Gaillet, Lise Musset, Claire Cropet, Félix Djossou, Adeline Mallard, Guillaume Odonne, Damien Davy, Maylis Douine, Loic Epelboin, Yassamine Lazrek, Luana Mathieu, Mathieu Nacher, Emilie Mosnier

**Affiliations:** 1 Service des Centres Délocalisés de Prévention et de Soins, Centre Hospitalier Andrée Rosemon, Cayenne, French Guiana France; 2 Écosystèmes Amazoniens Et Pathologie Tropicale, EA3593, Université de Guyane, Cayenne, French Guiana France; 3 Laboratoire de Parasitologie, Centre National de Référence du Paludisme, Pôle Zones Endémiques, WHO Collaborating Center for Surveillance of Antimalarial Drug Resistance, Institut Pasteur de La Guyane, Cayenne, French Guiana France; 4 Centre d’investigation Clinique, INSERM1424, Centre Hospitalier Andrée Rosemon, Cayenne, French Guiana France; 5 Unité de Maladies Infectieuses et Tropicales, Centre Hospitalier Andrée Rosemon, Cayenne, French Guiana France; 6 UMSR Laboratoire Écologie, Évolution, Interactions des Systèmes Amazoniens, CNRS-Université de Guyane-IFREMER, OHM Oyapock, Cayenne, French Guiana France; 7 Aix Marseille Univ, INSERM, IRD, SESSTIM, Sciences Economiques & Sociales de La Santé & Traitement de L’Information Médicale, ISSPAM, Marseille, France; 8 ANRS, MIE, University of Health Sciences, Phnom Penh, Cambodia

**Keywords:** Malaria, Knowledge, Attitudes and practices survey, French Guiana, Amazonia, Border, Malaria elimination

## Abstract

**Background:**

A steady decline in the number of cases of malaria was observed in the 2000s in French Guiana. This enabled regional health policies to shift their public health goal from control to elimination. To include inhabitants in this strategy, the main objective of this study was to describe knowledge about malaria, and related attitudes and practices in persons living in the French Guiana border.

**Methods:**

We conducted a survey in people over 15 years old living in the twelve neighbourhoods of Saint-Georges de l’Oyapock with the highest malaria incidence. It comprised a 147-item questionnaire which collected data on socio-demographic characteristics and included a Knowledge Attitude and Practices survey on malaria. Knowledge-related data were studied using exploratory statistical methods to derive summary variables. A binary variable assessing level of knowledge was proposed and then assessed using exploratory approaches.

**Results:**

The mean age of the 844 participants was 37.2 years [15.8], the male/female sex ratio was 0.8. In terms of nationality, 485 (57.5%) participants were Brazilian and 352 (41.7%) French. One third (305, 36.1%) spoke Brazilian Portuguese as their native language, 295 (34.9%) the Amerindian language Palikur, 36 (4.3%) French. The symptoms of malaria and prevention means were poorly known by 213 (25.2%) and 378 (44.8%) respondents, respectively. A quarter (206, 24.4%) did not know that malaria can be fatal. Overall, 251 people (29.7%) had an overall poor level of knowledge about malaria. Being under 25 years old, living in a native Amerindian neighbourhood, having an Amerindian mother tongue language, having risk behaviours related to gold mining were significantly associated with a poor level of knowledge.

**Conclusions:**

This study is the first to describe the poor level of knowledge about malaria in populations living in the malaria endemic border area along the Oyapock river in French Guiana. Results will allow to reinforce, to diversify and to culturally adapt prevention messages and health promotion to increase their effectiveness with a view to quickly reaching the goal of malaria elimination through empowerment.

**Supplementary Information:**

The online version contains supplementary material available at 10.1186/s12889-023-16507-9.

## Background

In 2022, France committed to eliminating malaria by 2025 in its overseas region of French Guiana. In fact, after a general incidence around 3.5% in the 90’s, this parameter reaches 0.01% in 2022 [1]. *P. vivax* is the predominant species if not exclusive in certain areas. Few cases of *P. falciparum* are observed with a majority of imported cases from West Africa [1]. Malaria transmission can nowadays be divided into two major transmission settings: the deep forest where gold mining activities occur [2, 3], and along the upper side of the Oyapock river at the border with Brazil [4]. Transmission also occurs sporadically in the west along the Maroni River, at the border with Suriname. The Oyapock and Maroni rivers represent borders but are also two major life basins with substantial cross-border movement of people [5].

The malaria program has to be adapt to elimination, but nowadays, malaria control includes free and passive diagnosis and treatment associated with bed-net distribution for pregnant woman [6]. Care delivering is organized around medical activities as community health worker are not authorize to diagnose and treat malaria in France. Outside the coastal area where biological laboratories and general medical practitioners are located, a network of health centres deliver cares. Some people live at several hours by foot or boat from it. Regarding treatment, radical cure for *P. vivax* includes a G6PD deficiency testing before primaquine prescription. As this biological exam is realized in France mainland, primaquine coverage is limited and relapses occurred before drug intake [2]. Since 2015, operational research programmes aimed to test new approaches to allow an evidence-based elaboration of the future elimination program [7]. The MALAKIT program tested a self-diagnosis and self-treatment approach for gold miners [8] while, ELIMALAR/PALUSTOP aimed to evaluate the performances of a mass-screen and treat approach in the specific key populations of the Upper Oyapock area [4].

Malaria elimination requires a huge level of involvement from all the stakeholders implicated, including authorities, healthcare professionals, and populations living in endemic areas. The success of elimination in French Guiana will depend on the capacity of the territory, to deploy prompt diagnosis and treatment in these key specific population living in hard-to-reach mining areas or remote villages. It will also require empowering all the people of this culturally diverse and mixed population for malaria prevention using adapted education tools.

In this context, assessing knowledge about malaria, related attitudes, and associated practices in at-risk populations living in endemic foci is essential to identify groups of individuals requiring educational actions, and to ensure that the educational tools implemented are culturally appropriate. No data are available regarding knowledge, attitudes, and practices (KAP) of local population living outside the gold mining camps [9] except practices regarding the use of plants as traditional remedies [10]. In the Amazonian context, some studies have investigated these aspects [11–15] however, results are generally specific of an area and population.

The main objective of the present study was to describe malaria knowledge in persons living in the key epidemic focus area of Saint Georges de l’Oyapock, at the border between French Guiana and Brazil.

## Methods

### Design of the study

This cross-sectional study was conducted between September and December 2017, during the first phase of the "before-after" interventional project called "PALUSTOP" [4]. It consisted of a Knowledge Attitudes and Practices (KAP) survey carried out at the time of inclusion of Saint-Georges neighbourhood residents, i.e. in the "pre-intervention" phase, using a questionnaire that also collected socio-demographic data.

A mobile team comprising community workers, physicians and nurses oversaw participant inclusion. In order to guarantee standardized implementation, the community workers were trained on administering the study questionnaire prior to the study. Questionnaire items were asked in the native language of the participants.

### Population and study area

**Fig. 1:**
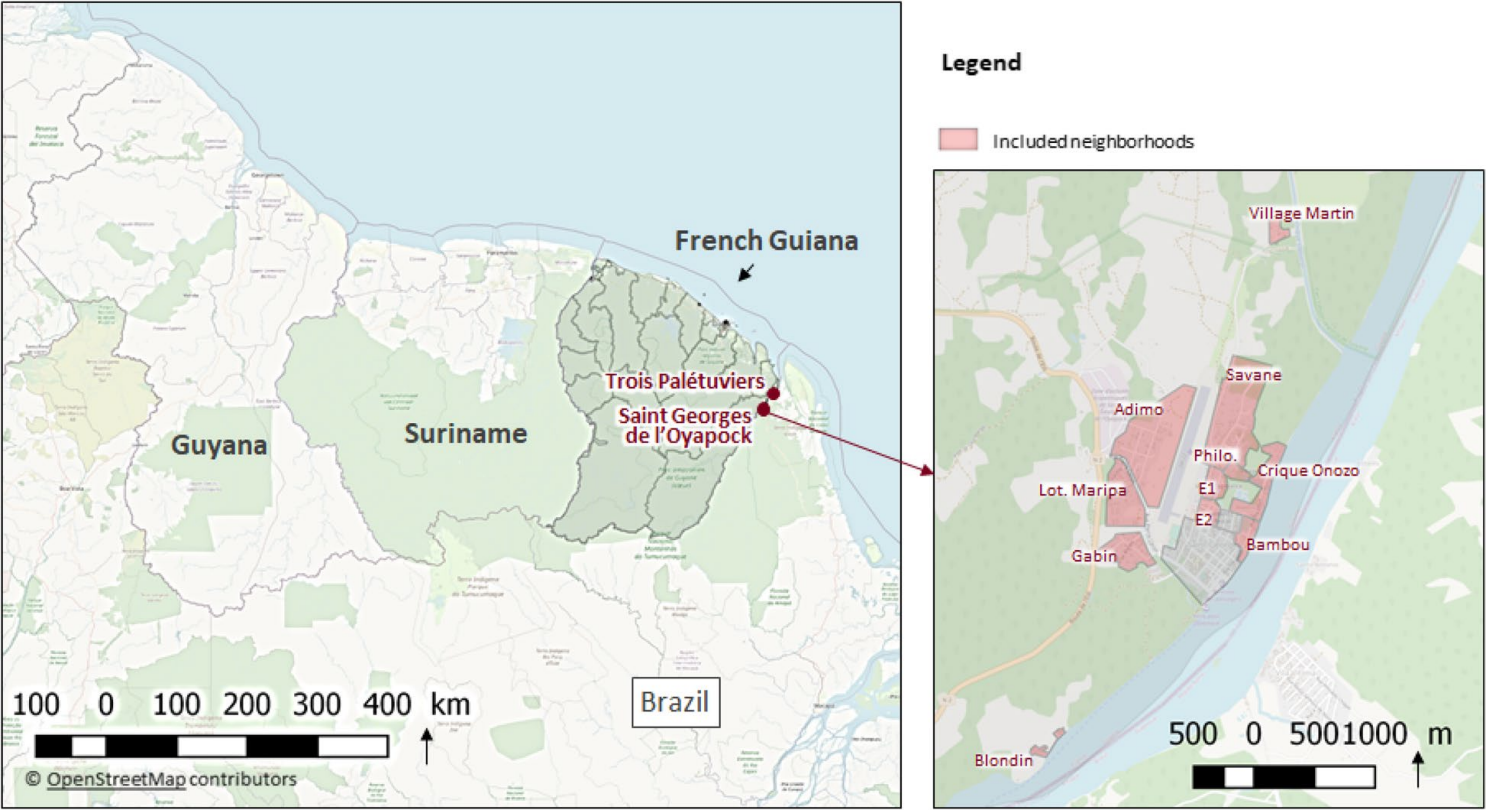
The neighbourhoods of Saint Georges de l’Oyapock municipality targeted by the KAP study

The municipality of Saint Georges de l’Oyapock is located along the Oyapock River in the Amazonian forest, 200 km from Cayenne, the administrative main city of French Guiana (see Fig. 1). On the other side of the river is the Brazilian municipality of Oiapoque. Saint Georges de l’Oyapock has approximately 4,020 inhabitants from different communities: Amerindians, Brazilians, Creoles, Europeans and Maroons (https://www.insee.fr/fr/statistiques/6681450) [4]. This municipality is located in a malaria transmission area with a seasonal transmission occurring mainly between September and January each year. In 2017, malaria cases recorded in St Georges de l’Oyapock represented 50% of the total cases registered in French Guiana. Considering the inhabitants exposed to malaria in the 12 neighbourhoods experiencing transmission, malaria incidence was of 2.0%, 7.9%, 4.1% and 1.7% in 2016, 2017, 2018 and 2019, respectively. Almost all the cases are due to *P. vivax* and a large part are related to relapses because of a low coverage of primaquine [2].

This KAP survey included individuals over 15 years old of 12 out of 16 neighbourhoods in Saint Georges de l’Oyapock. These neighbourhoods were chosen study because they recorded the highest malaria incidence in the three preceding years. They accounted for 82% of the municipality’s population (*n* = 3,307). This KAP study was a part of a large mass screen and treat intervention named “PALUSTOP” [4]. Participants answered the questionnaire during the first intervention in 2017. They were not sampled because the objective was to be as exhaustive as possible for mass malaria screening and treatment.

### KAP survey and collection of socio-demographic data

The survey questionnaire comprised 147 items which collected socio-demographic data and KAP data. More specifically, eight items concerned socio-demographic information and six concerned sources of information on malaria. With respect to the KAP items nested in the larger questionnaire, six items concerned knowledge about the disease, while a total of 49 concerned associated attitudes and related practices. The questionnaire’s other 78 items focused on clinical, ethno-botanical and biological data (not shown). The present study only describes the KAP analysis; sociodemographic, clinical, and biological dimensions are not described.

## Statistical analysis

### Construction of variables

To construct the variables about patient knowledge, we explored, i) data using statistical methods as flat and spherical principal component analyses (PCA), focused PCA, correlation matrices, and factorial analyses (Appendices 1 and 2) and, ii) the literature on this topic [11–15].

For the six explored themes, knowledge about the disease’s symptoms, transmission, prevention, and treatment were evaluated based on multiple-choice questions. Each response proposal was recoded into as many binary outputs (Yes/No). A score has been assigned to each of them when they were considered correct or not (See Table 1). According to them, “good”, “insufficient” or “bad” knowledge categories have been constructed using the different variables listed in Table 1.

**Table 1:** Variables and knowledge about malaria, Saint Georges de l’Oyapock, 2017 (*n* = 844 respondents)

	Answer Affected score	Good answer proportion Number of respondent (%)
**Knowledge of symptoms**		
*** Headache***	1	479 (56.8)
Fever	1	393 (46.6)
Body ache	1	303 (35.9)
Chills	1	256 (30.3)
Tiredness	1	174 (20.6)
Abdominal pain	1	145 (17.1)
Other responses	0	141 (16.7)
Do not know	0	91 (10.8)
Diarrhoea	1	62 (7.3)
Jaundice	1	2 (0.2)
***Good knowledge of symptoms***	> 3/10	221 (26.2)
***Insufficient knowledge of symptoms***	1/10 to 3/10	434 (40.6)
***Poor knowledge of symptoms***	0/10	213 (25.2)
**Knowledge of transmission mode**		
*** Mosquitoes***	Yes	617 (73.1)
Do not know	0	92 (10.9)
Drinking water	0	21 (2.5)
Other responses	0	15 (1.8)
Air	0	6 (0.7)
People	0	1 (0.1)
Food	0	0 (0.0)
***Good knowledge of transmission mode***	Mosquitoes yes + ≤ 1 incorrect	592 (70.1)
***Insufficient knowledge of transmission mode***	Mosquitoes yes + 2 ou 3 incorrect	27 (3.2)
***Poor knowledge of transmission mode***	Mosquitoes no ± other answers or Mosquitoes yes + ≥ 4 incorrect	227 (26.9)
**Knowledge of prevention means**		
Mosquito net	3	408 (48.3)
Don’t know	0	257 (30.5)
Cutaneous repellent	1	149 (17.7)
Empty water containers	0	144 (17.1)
Repellent spray or spiral	1	100 (11.8)
Other responses	0	71 (8.4)
Long clothing	1	59 (7.0)
Drugs	3	53 (6.3)
Plants	0	50 (5.9)
Outdoor spraying	2	21 (2.5)
Fans	0	17 (2.0)
None	0	12 (1.4)
***Good knowledge of prevention means***	> 5	335 (42.1)
***Insufficient knowledge of prevention means***	3 ≤ points < 5	111 (13.2)
***Poor knowledge of prevention means***	< 3	378 (44.8)
**Knowledge of therapeutic means**		
Drugs	Yes	623 (73.8)
Do not know	0	166 (19.7)
Plants	0	148 (17.5)
None	0	14 (1.7)
Other responses	0	11 (1.3)
***Good knowledge of therapeutic means***	*Drugs = yes*	60.5% (511)
***Insufficient knowledge of therapeutic means***	Drugs = yes + incorrect answers	112 (13.3)
***Poor knowledge of therapeutic means***	Drugs = no	221 (26.2)

In addition to these four themes, the evaluation of the general knowledge about malaria was based on two questions, “Have you ever heard of malaria?” and “Can you die from malaria?” and their binary answers, “yes” or “no”.

A poor level of knowledge of malaria was assigned for people who answered:

i) "no" to the question "Have you ever heard of malaria?",
ii) "yes" but met at least two out of four conditions regarding the thematic knowledge: symptoms, answer “don’t know”; transmission mode, mosquitoes was not identified; prevention means, no answer; "Can you die from malaria?", answer “no”.

All those who did not have a poor level of knowledge about malaria were considered to have a good level of knowledge.

### Statistical methods

We used Student’s t-test to compare quantitative variables, and the Chi2 test for qualitative variables. In case of non-compliance with the validity conditions set out for each test, Wilcoxon’s (or Kruskal–Wallis if more than two classes) or Fisher’s (or an ANOVA if more than 2X2 tables) nonparametric tests were used. The significance level used for the interpretation of the statistical tests was set at *p* < 0.05.

Data were analysed using R software, version 3.4.2.

### Ethical approval and consent to participate

The studies on the patients' biological samples were performed in accordance with the relevant guidelines and regulations. The study was approved by the French ethics committee of protection of persons of south-west and overseas 4 N° AM-36/1/CPP15-024.

The database was anonymized and declared to the French Regulatory Commission (Commission Nationale Informatique et Libertés, CNIL, n°917,186). Signed informed consent was obtained from all participants involved in the study, following explanations about the study in their native language. For minor patients, informed consent was obtained from a parent and/or legal guardian. Patients who did not received formal education were illiterate. However, illiteracy did not deprive them of the ability to give or withhold informed consent. It was collected.

## Results

A total of 844 individuals over 15 years old participated in the KAP study and answered the questionnaire. The average age of the respondents was 37.2 years [15.8], and the male/female sex ratio was 0.8 (Table 2). A majority, 57.5% (*n* = 485) were Brazilian nationals. Two thirds, 67.5% (*n* = 575) had a history of malaria. A, quarter 20.8% (*n* = 260) had a primary school education level or  had never attended school. One in seven, 14.2% (*n* = 120) did not have any social security cover, while 6.5% (*n* = 55) had standard social cover.

**Table 2:** Bivariate study of sociodemographic variables according to poor or good knowledge, St Georges de l’Oyapock, 2017

	**Characteristics of the study population**	**Poor knowledge**	**Good knowledge**	**p-value**
**N (%) / mean (standard deviation)**	844 (100)	251 (28.7)	593 (71.3)	
**Gender** **(M / F)**	363 (42.6) / 481 (57.4)	107 (42.6) / 144 (57.4)	256 (43.2) / 337 (56.8)	0.945
**Age (years)**	37.2 (15.8)	37.5 (18.7)	37.1 (14.5)	0.204
15 – 24	225 (26.7)	80 (31.9)	145 (24.5)	0.013
25 – 49	448 (53.1)	114 (45.4)	334 (56.3)	
≥ 50	171 (20.3)	57 (22.7)	114 (19.2)	
**Length of stay at Saint Georges (years)**	17.8 (14.0)	19.6 (15.8)	17.0 (13.0)	0.014
**Number of people in household**	6.9 (3.2)	7.1 (3.4)	6.8 (3.1)	0.217
**History of malaria** **Yes (%)**	570 (67.5)	126 (50.2)	444 (74.9)	< 0.001
**Number of malaria attacks**	24 (4.9)	1.1 (1.6)	3.0 (5.7)	< 0.001
**Neighbourhood**				< 0.001
Savane	230 (27.3)	56 (22.3)	174 (29.3)	
Onozo	144 (17.1)	29 (11.6)	115 (19.4)	
Trois-Palétuviers	83 (9.8)	28 (11.2)	55 (9.3)	
Espérance2	75 (8.9)	38 (15.1)	37 (6.2)	
Adimo	62 (7.4)	24 (9.6)	38 (6.4)	
Gabin	57 (6.8)	14 (5.6)	43 (7.3)	
Espérance1	51 (6.0)	22 (8.8)	29 (4.9)	
Philogène	42 (5.0)	16 (6.4)	26 (4.4)	
Blondin	30 (3.6)	3 (1.2)	27 (4.6)	
Maripa	27 (3.2)	7 (2.8)	20 (3.4)	
Bambou	22 (2.6)	5 (2.0)	17 (2.9)	
Village Martin	21 (2.5)	9 (3.6)	12 (2.0)	
**School level**				< 0.001
No formal education^1^	116 (3.7)	61 (24.3)	55 (9.3)	
Primary	144 (17.1)	49 (19.5)	95 (16.0)	
Lower secondary school	341 (40.4)	84 (33.5)	257 (43.3)	
Upper secondary school	202 (23.9)	53 (21.1)	149 (25.1)	
University	41 (4.9)	4 (1.6)	37 (6.2)	
**Nationality**				0.171
Brazilian	485 (57.5)	134 (53.4)	351 (59.2)	
French	352 (41.7)	116 (46.2)	236 (39.8)	
Other	7 (0.8)	1 (0.4)	6 (1.0)	
**Native language**				< 0.001
Brazilian Portuguese	305 (36.1)	39 (15.5)	266 (44.9)	
Palikur	222 (26.3)	101 (40.2)	121 (20.4)	
Creole	168 (19.9)	64 (25.5)	104 (17.5)	
Karipuna	49 (5.8)	16 (6.4)	33 (5.6)	
French	36 (4.3)	4 (1.6)	32 (5.4)	
Wayampi, Kalina, Teko	24 (2.8)	13 (5.2)	11 (1.9)	
Other	40 (4.7)	14 (5.6)	26 (4.4)	
**Social security cover**				0.295
Universal health cover^2^	531 (63.0)	161 (64.1)	370 (62.4)	
None	120 (14.2)	43 (17.1)	77 (13.0)	
State Medical Assistance^3^	73 (8.7)	16 (6.4)	57 (9.6)	
Standard social cover	55 (6.5)	12 (4.8)	43 (7.3)	
Brazilian social rights	43 (5.1)	12 (4.8)	31 (5.2)	
Do not know	22 (2.6)	7 (2.8)	15 (2.5)	
**Occupation**				< 0.001
Unemployed	310 (36.7)	97 (38.6)	213 (35.9)	
Other	179 (21.2)	36 (14.3)	143 (24.1)	
Farmer	108 (12.8)	43 (17.1)	65 (11.0)	
Employee	92 (10.9)	15 (6.0)	77 (13.0)	
Student	54 (6.4)	22 (8.8)	32 (5.4)	
Hunter	37 (4.4)	12 (4.8)	25 (4.2)	
Retired	36 (4.3)	21 (8.4)	15 (2.5)	
Fisherman	28 (3.3)	5 (2.0)	23 (3.9)	
**Number of sources of information about malaria**		0.6 (0.8)	1.6 (1.0)	< 0.001
**Sources of information Yes (%)**				
School	123 (14.6)	28 (11.2)	95 (16.0)	0.085
Physician	314 (37.2)	38 (15.1)	276 (46.5)	< 0.001
Family	309 (36.6)	54 (21.5)	255 (43.0)	< 0.001
Poster	23 (2.7)	1 (0.4)	22 (3.7)	0.005
Friend	126 (14.9)	11 (4.4)	115 (19.4)	< 0.001
Journal	19 (2.2)	1 (0.4)	18 (3.0)	0.020
Radio	25 (3.0)	3 (1.2)	22 (3.7)	0.072
Television	101 (12.0)	19 (7.6)	82 (13.8)	0.015
Other	88 (25.6)	7 (2.8)	81 (13.7)	< 0.001
**Do you think you have enough information about the definition of malaria?**				< 0.001
Yes (n/%)	377 (44.7)	39 (15.5)	338 (57.0)	
No (n/%)	345 (40.9)	90 (35.9)	255 (43.0)	
Do not know (n/%)	122 (14.5)	122 (48.6)	0 (0.0)	
**Do you think you have enough information about what to do in case you get malaria?**				< 0.001
Yes (n/%)	461 (54.6)	60 (23.9)	401 (67.6)	
No (n/%)	260 (30.8)	68 (27.1)	192 (32.4)	
Do not know (n/%)	123 (14.6)	123 (49.0)	0 (0.0)	
**Do you think you have enough information about how to prevent getting malaria?**				< 0.001
Yes (n/%)	334 (39.6)	34 (13.5)	300 (50.6)	
No (n/%)	388 (46.0)	95 (37.8)	293 (49.4)	
Do not know (n/%)	122 (14.5)	122 (48.6)	0 (0.0)	

^1^These patients were illiterate. However, illiteracy did not deprive them of the ability to give or withhold informed consent. It was collected

^2^Universal health coverage: Universal protection against illness provided to people with very low income

^3^State Medical Assistance: Social security cover for immigrants with no residency permit or a document proving he/she has begun the application process for legal residency

The main occupations were rural in nature (hunting, fishing, or agriculture), totalling 20.5% of respondents (*n* = 173), while 41.0% (*n* = 346) were unemployed or were retired.

**Fig. 2:**
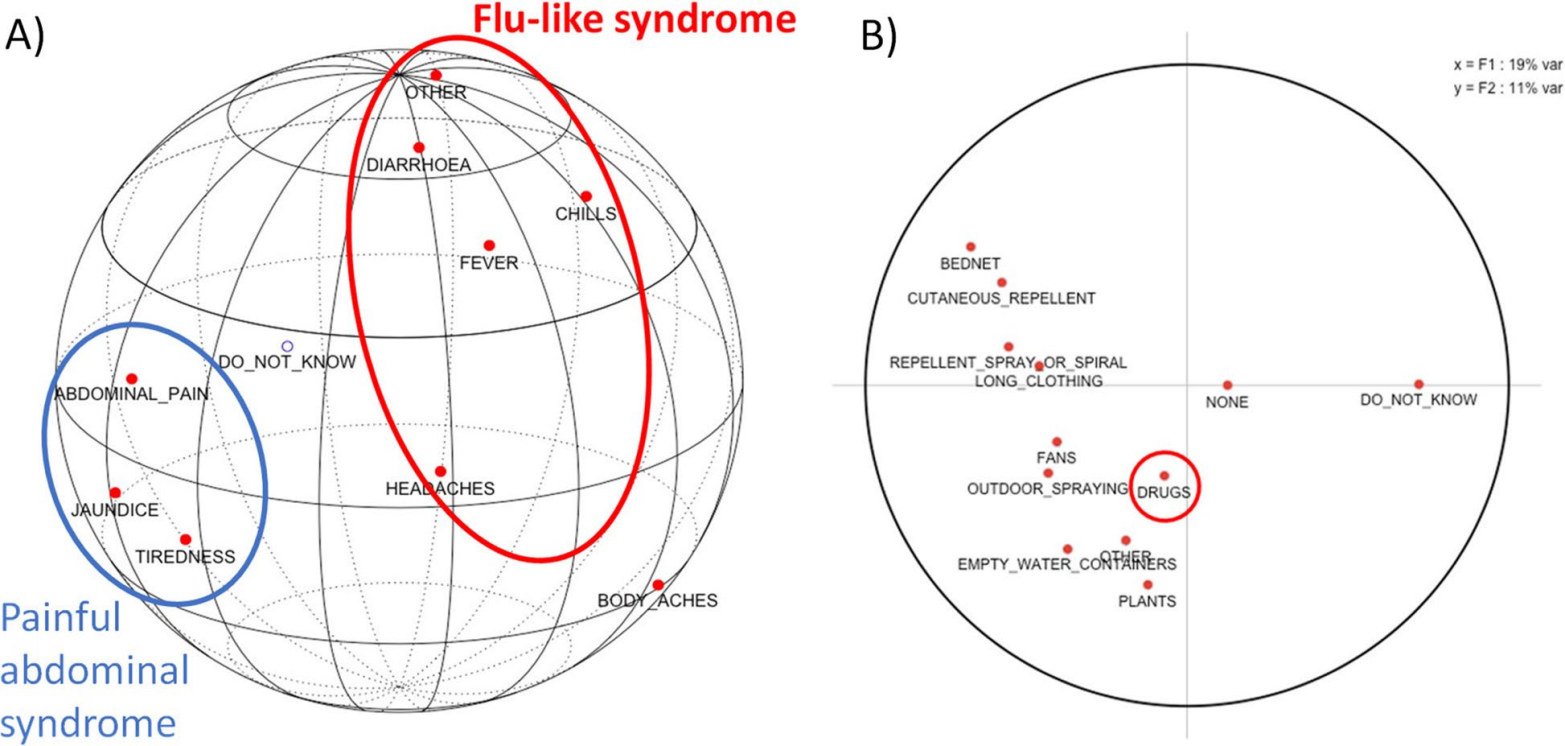
Exploratory analysis of multiple-choice questions to assess inhabitant’s knowledge of malaria, **A**) symptoms and **B**) prevention means. Interpretation of a spherical principal component analysis (**A**): when two variables are close to each other they are strongly correlated. When two variables are separated from the centre by a 90 degrees angle then they are almost independent. When two variables are diametrically opposed, then they are correlated a priori negatively. Interpretation of a flat principal component analysis (**B**): when two points are close to the edge of the circle and are close to each other, then the two underlying variables are also correlated. When there are two points close to the circle and diametrically opposed, then the underlying variables are negatively correlated. When two points are close to the circle and at right angles to the origin, then the underlying variables are uncorrelated. As soon as points are removed from the circumference of the circle, we can no longer interpret the results of a principal component analysis

Only a quarter (*n* = 221, 26.2%) had good knowledge about the symptoms of malaria (Table 1). Headache and fever were identified respectively by 56.8% (*n* = 479) and 46.6% (*n* = 393) of the respondents. Nevertheless, 27.5% (*n* = 232) did not know or cited wrong symptoms of the disease. The different ‘symptom’ variables were very weakly associated with each other (Fig. 2A). However, factorial analysis (Appendices 1 and 2) showed that the study population identified two syndromes: a flu-like syndrome (headache, chills, body aches, fever) and a painful abdominal syndrome (asthenia and abdominal pain).

Mosquitoes were recognized as a vector for malaria by 73.1% (*n* = 617) of participants. However, only 48.3% (*n* = 408,) mentioned mosquito bed nets as a preventing mean even if this prevention tool was considered apart from the others prevention means by respondents (Fig. 2B). Globally, prevention means were poorly known (44.8%, *n* = 378) with 31.9% (*n* = 269) of the respondents who could not identified any of them.

Regarding treatment, 26.2% (*n* = 221) did not know that malaria could be cured by drugs and drugs as a chemoprophylaxis were not associated by the participants with malaria prevention (Table 1, Fig. 2B).

The overall level of knowledge about malaria was ‘good’ for 71.3% (*n* = 593) of the participants (Table 2). Those people were more likely to be employed, fishermen (p < 0.001), to already have had malaria (*p* < 0.001), and to have suffered from a greater number of malaria attacks than those having a ‘poor’ level of knowledge (3.0 vs 1.1, *p* < 0.001). People who declared having physicians, friends, and family as sources of information about malaria had also a better level of knowledge (*p* < 0.001). Moreover, the higher the number of information sources, the better was respondents’ level of knowledge (0.6 vs 1.6; *p* < 0.001).

A quarter, 24.4% (*n* = 206) did not know that malaria can be fatal. A minority of participants, 14.4% (*n* = 122), had no knowledge of malaria. People with a “poor” knowledge of malaria were significantly more likely to be under 25 years old (*p* = 0.013) and to have only primary education or no formal education (*p* < 0.001) (Table 2). A third of the people with a ‘poor’ level of knowledge thought that the level of information available on malaria was insufficient in terms of its definition (35.9%), good practices to adopt when infected (27.1%), or to prevent malaria (37.8%).

The analysis of the level of knowledge according to a participant’s native language highlighted that those who spoke Brazilian Portuguese had a better level of knowledge than those whose mother tongue was the Amerindian language Palikur (*p* < 0.001).

In terms of practices, 74.2% (*n* = 626) of the respondents mentioned they slept under a mosquito bed net to prevent infection whatever their level of knowledge about malaria (Table 3). Bed nets were not impregnated with insecticide for 23.9% (*n* = 202) of the people and around the same proportion had holes on it (30.8%, *n* = 260).

**Table 3:** Bivariate study of attitudes and practices variables according to ‘good’ or ‘poor’ knowledge

	**Poor knowledge**	**Good knowledge**	***p*-value**
**N (%) / mean (standard deviation)**	251	593	
**Do you have a mosquito net over your bed or over your windows?** **Yes (%)**	14 (5.6)	55 (9.3)	0.098
**Do you sleep under a mosquito bed net? Yes (%)**	202 (80.5)	424 (71.5)	0.008
**How old is your mosquito bed net (years)?**	0.51 (0.5)	0.44 (0.5)	0.070
**Is your bed net impregnated with insecticide?**			0.062
Yes	120 (47.8)	289 (48.7)	
No	72 (28.7)	130 (21.9)	
Do not know	59 (23.5)	174 (29.3)	
**Does your bed net have holes?**			0.011
Yes	78 (31.1)	182 (30.7)	
No	123 (49.0)	238 (40.1)	
Do not know	50 (19.9)	173 (29.2)	
**When you have a fever or when you had malaria, do or did you consult a physician?**			0.043
Never	13 (5.2)	32 (5.4)	
Sometimes	97 (38.6)	283 (47.7)	
Often	141 (56.2)	278 (46.9)	
**Do you use individual cutaneous repellents?**			0.295
Never	204 (81.3)	456 (76.9)	
Sometimes	39 (15.5)	107 (18.0)	
Often	8 (3.2)	30 (5.1)	
**Do you use insecticides for the house (spray cans or spirals)?**			0.031
Never	87 (34.7)	156 (26.3)	
Sometimes	142 (56.6)	364 (61.4)	
Often	22 (8.8)	73 (12.3)	
**Have you taken a medicine against malaria without a prescription?** **Yes (%)**	219 (87.3)	487 (82.1)	0.082
**Do you use plants to treat malaria? Yes (%)**	209 (83.3)	515 (86.8)	0.210
**Risk-taking related to gold mining** **Yes (%)**	224 (89.2)	491 (82.8)	0.023
**Hunting**			0.622
Never	188 (74.9)	447 (75.4)	
Sometimes	54 (21.5)	117 (19.7)	
Often	9 (3.6)	29 (4.9)	
**Fishing**			0.677
Never	165 (65.7)	374 (63.1)	
Sometimes	69 (27.5)	181 (30.5)	
Often	17 (6.8)	38 (6.4)	
**Do you go into the rain forest?**			0.271
Never	116 (46.2)	301 (50.8)	
Sometimes	85 (33.9)	199 (33.6)	
Often	50 (19.9)	93 (15.7)	

In case of fever, 5.3% (*n* = 45) of the respondent never consult a physician and 45.0% (*n* = 380) only sometimes. Auto medication is the rule (83.6%, *n* = 706), essentially with plants. The level of knowledge of malaria do not influence this practice (87.3% vs 82.1%, *p* = 0.082).

Finally, people who had links with gold mining (i.e., working there or hosting people from those settings) had “poor” knowledge about malaria (89.2%* vs *82.8%*, p = 0.023).*

## Discussion

This study identified a poor level of knowledge about malaria among 29.7% (*n* = 251) of the respondents despite an history of malaria for 67.5% of them. This poor level of knowledge was more likely observed in people under 20 years old, or living in a peripheral neighbourhood of the municipality, or speaking an Amerindian language. People mostly protected themselves using mosquito bed nets and at a lower level, domestic insecticides.

The knowledge of malaria symptoms was poor or insufficiently known for 65.8% of the respondents and headaches (56.8%) more cited than fever (46.6%). In similar epidemiological context, 52.4% of the inhabitants identified fever in Colombia [13] and as low as 12.8% in Venezuela [14]. In Africa where transmission levels are high and *P. falciparum* predominant, knowledge about symptoms are clearly higher, 82.8% (*n* = 1,617) [16]. This situation in South America may be explained by the fact that *P. vivax* infections are predominant. With this species, malaria symptomatology is particularly non-specific and recurrence of attenuated forms is frequent [17]. The ‘normalization’ of malaria, described recently in the Amazonian population in Peru can explain this lack or insufficient perception of symptoms in case of *vivax*-malaria [12]. Furthermore, Amerindian populations’ representations of fever and malaria differ widely from Western representations. To the former, diseases are seen as an imbalance of nature, a crisis with different causes. Shamans play an important role in this representation [18]. The health education messages based on symptom identification need to consider these cultural differences to be effective. This underscores the crucial role of health mediation in multi-ethnic territories.

Mosquitoes were identified as the vector by two-third of the respondents. Only half of them cited bed nets as a mean of prevention but 74.2% (*n* = 626) slept under them even more if they have a poor level of knowledge about malaria. The exploratory statistical analyses confirmed that people identified bed nets as a prevention tool which was different from others. The large distribution and education campaign around bed nets done few years ago could explain this observation [19].

The use of drugs to prevent malaria was also poorly known by patients but the absence of preventive treatment in French Guiana could explain this situation [6]. Surprisingly, outdoor spraying in villages was cited by only 21 people (2.5%), despite the fact that regular insecticide campaigns have been taking place in French Guiana since the 1950s [2, 20]. This suggests that people participating in the study did not understand that it was a tool against malaria. Globally, knowledge regarding prevention means were poor for 44.8% (*n* = 378). The use of prevention methods and adherence to malaria treatment are closely linked to the perception of the risk of mortality [21]. However, 24.4% (*n* = 206) of the respondents did not know that malaria can be fatal.

Drugs to treat malaria were not identified by 26.7% (*n* = 221) of the respondents. In French Guiana, primaquine coverage is very low and patients generally experience relapses [2, 4, 18]. This specific situation may have contributed to participants’ beliefs that there is no treatment for malaria and explain why knowledge level of therapeutic means was much lower (73.8%, *n* = 623) than that commonly observed in other malaria endemic areas, 89.3% in Colombia [13] or 93.2% in India [22]. Knowing that malaria can be cured is essential to limit transmission and at the end, eliminate the disease [23]. A vast majority of the respondents used plants to treat malaria (85.8%, *n* = 724) whatever their level of knowledge regarding malaria (*p* = 0.21). A substantial proportion of the respondents (16.5%, *n* = 139), considered plants as a therapeutic means. This demonstrates the widespread use of traditional medicines in French Guiana, whatever the community [24, 25] and highlights the importance of a better understanding of traditional medicine to improve adherence to drugs and acceptability of non-traditional antimalarial treatments. To achieve malaria elimination, traditional and non-traditional medicine should be considered as complementary and not opposed treatments.

We were able to identify specific groups whose level of general knowledge about malaria was poor. As in other settings, young people were identified in this category [26]. Living in the two peripheral neighbourhoods of the Saint Georges de l’Oyapock municipality, Blondin and Trois Palétuviers, was also a factor of poor general knowledge about malaria, as well as those whose native language was an Amerindian language (vs. Brazilian Portuguese and French-speaking persons, *p* < 0.001). These three characteristics were previously observed in 2012 in an assessment of HIV knowledge of a population of lower secondary school students in the municipality [27]. One explanation for these similar results is that most of the information/education system processes in French Guiana are in French and Brazilian Portuguese and take a European approach to delivering disease education messages. The Brazilian malaria prevention programme seems to be more effective. The regional health authorities in French Guiana could draw inspiration from it in the context of cross-border cooperation.

As previously described in the literature, results shown that having a low level of education coupled with high economic insecurity (i.e., a large number of people per household, poor social security cover, unemployment) was associated with a poor level of knowledge about malaria [27]. Conversely, a higher level of education significantly improves knowledge, which itself increases the adoption of preventive measures for malaria, without affecting the perception of risk [28].

This part of French Guiana is a transborder area where people are living/moving between Brazil and French Guiana and are of mixed and multi-cultural origins talking several languages. This KAP study was not implemented to characterize ethnic groups and their specific representations. Consequently, we are not able to identify and characterize subpopulations satisfactorily for each level of knowledge of malaria. In addition, the relatively small number of respondents limited the study power when results and sub-categorized.

Our study highlights that participants were open to receiving more information about the disease and how to prevent it (…). Therefore, if we want to eliminate malaria, health promotion tools should be elaborated considering the different cultural specificities of the population of this area as a whole to empower all individuals and communities [28–30]. The development of cultural mediation in health could be an effective means to implement adapted and multilanguage prevention messages. The mobile public health teams recently implemented in French Guiana at the borders and in isolated areas [31], comprising nurses and mediators from the communities, offer a ‘going toward’ model of health promotion, which could partly meet the needs identified in this study.

## Conclusion

This study, conducted in French Guiana, provides information for an evidence-based malaria prevention program and gives perspectives in terms of health education. Health promotion tools should be implemented to at-risk populations with a poor level of knowledge about malaria in this endemic area. Valorising autochthonous traditional care practices and considering local representations of the disease as a complement to biomedical approaches are important actions. Health mediation would appear to be essential to conduct such actions, specifically to increase the level of health literacy of key populations so that they can in turn participate in achieving the objective of eliminating malaria in French Guiana by 2025.

### Supplementary Information

**Additional file 1:** **Appendix 1.** Factor analyses of multiple-choice variables assessing knowledge of symptoms.

**Additional file 2:** **Appendix 2.** Factor analyses of multiple-choice variables assessing knowledge of malaria prevention methods

## Data Availability

The datasets generated and analysed in the present study are not publicly available, as special authorization is required to transfer databases provided by the CNIL. With prior CNIL authorization, the datasets can be made available from the corresponding author upon reasonable request.

## Abbreviations

ANOVA: Analysis of variance
KAP: Knowledge Attitudes and Practices (survey)
PCA: Flat and spherical Principal Component Analysis
PCR: Polymerase Chain Reaction
