# Supplementary material for: Determination of different social groups’ level of knowledge about malaria in a multicultural Amazonian cross-border context
Source: BMC Public Health. 2023 Aug 19;23:1585. doi: 10.1186/s12889-023-16507-9 (PMC10439639; doi:10.1186/s12889-023-16507-9)
Supplement: Supplementary file 1 — Additional file 1: Appendix 1. Factor analyses of multiple-choice variables assessing knowledge of symptoms. [file 12889_2023_16507_MOESM1_ESM.docx]

**APPENDIX**

***Appendix 1. Factor analyses of multiple-choice variables assessing knowledge of symptoms***

| **Symptoms** | **Factor 1** | **Factor 2** |
| --- | --- | --- |
| **Tiredness** | 0.192 | 0.285 |
| **Headaches** | 0.587 | 0.361 |
| **Chills** | 0.388 | 0.112 |
| **Other** | 0.215 | 0.109 |
| **Body ache (not head)** | 0.434 | -0.060 |
| **Abdominal pain** | -0.007 | 0.762 |
| **Do not know** | -0.572 | -0.208 |
| **Diarrhoea** | 0.186 | 0.070 |
| **Fever** | 0.473 | 0.187 |
| **Jaundice** | 0.050 | 0.105 |

Interpretation of Factor Analysis: the objective of an FA is to determine which items belong to which dimension. From a formal point of view, an FA is a linear model that explains the responses to items using a small number of latent, unobserved variables.
