# Supplementary material for: Determination of different social groups’ level of knowledge about malaria in a multicultural Amazonian cross-border context
Source: BMC Public Health. 2023 Aug 19;23:1585. doi: 10.1186/s12889-023-16507-9 (PMC10439639; doi:10.1186/s12889-023-16507-9)
Supplement: Supplementary file 2 — Additional file 2: Appendix 2. Factor analyses of multiple-choice variables assessing knowledge of malaria prevention methods [file 12889_2023_16507_MOESM2_ESM.docx]

***Appendix 2. Factor analyses of multiple-choice variables assessing knowledge of malaria prevention methods***

|  | | | |
| --- | --- | --- | --- |
| **Prevention methods** | **Factor 1** | **Factor 2** | **Factor 3** |
| **Mosquito bed net** | 0.922 | 0.271 | -0.268 |
| **Cutaneous repellent** | 0.244 | 0.407 | 0.067 |
| **Drugs** | 0.017 | 0.013 | 0.302 |
| **Repellent sprays or spirals** | 0.178 | 0.446 | 0.040 |
| **Long clothing** | 0.061 | 0.411 | 0.123 |
| **Outdoor spraying** | 0.026 | 0.444 | 0.023 |
| **None** | -0.022 | -0.148 | 0.237 |
| **Plants** | -0.021 | 0.077 | 0.336 |
| **Fans** | 0.026 | 0.429 | 0.007 |
| **Empty water containers** | 0.146 | 0.214 | 0.280 |
| **Other** | 0.031 | 0.090 | 0.313 |
| **Do not know** | -0.842 | -0.163 | -0.509 |

Interpretation of Factor Analysis: the objective of an FA is to determine which items belong to which dimension. From a formal point of view, an FA is a linear model that explains the responses to items using a small number of latent, unobserved variables.
